# Supplementary material for: Association of Fish Oil Supplementation with Risk of Coronary Heart Disease in Individuals with Diabetes and Prediabetes: A Prospective Study in the UK Biobank
Source: Nutrients. 2023 Jul 17;15(14):3176. doi: 10.3390/nu15143176 (PMC10386642; doi:10.3390/nu15143176)
Supplement: Supplementary file 1 [file nutrients-15-03176-s001.zip › nutrients-2464217-supplementary.pdf]

Association of Fish Oil Supplementation with Risk of Coronary Heart Disease in Diabetes and Prediabetes Individuals: A Prospective Study

Xiaohui Liu, et al.

Figure S1. Flow chart of study participants 2

Table S1. β coefficients of fish oil supplement use for blood indicators from general linear regression analysis in the UK Biobank in participants with prediabetes and diabetes. 3

Table S2. Diet component definitions used in the UK Biobank study. 4

Table S3. Codes used in the UK Biobank to identify dementia cases and other relevant diseases. 5

Table S4. β coefficients of fish oil use for blood indicators from general linear regression analysis in the UK Biobank in participants with prediabetes and diabetes 6

Table S5. Sensitivity analyses for the HRs (95% CIs) of CHD according to fish oil supplement use among participants with prediabetes. 7

Table S6. Sensitivity analyses for the HRs (95% CIs) of CHD according to fish oil supplement use among participants with diabetes. 8

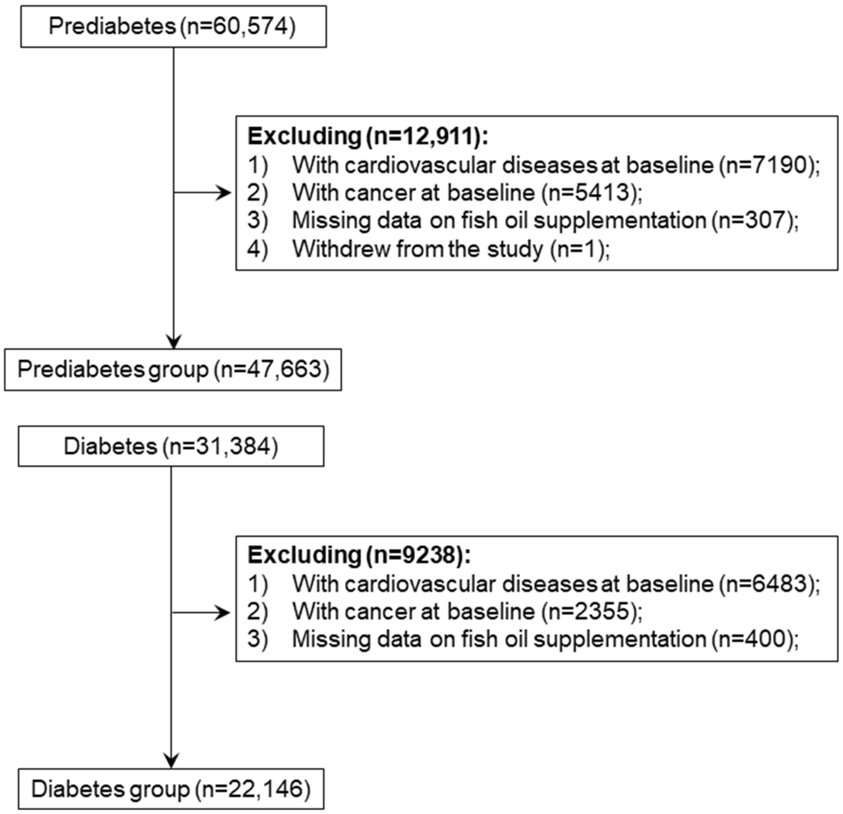

Figure S1. Flow chart of study participants

Table S1. β coefficients of fish oil supplement use for blood indicators from general linear regression analysis in the UK Biobank in participants with prediabetes and diabetes.

	Baseline	First repeat assessment (2012-2013)	Imaging visit (2014+)
**Prediabetes**			
No of participants	47,663	1705	3703
Baseline	1.00	0.61	0.49
First repeat assessment (2012-2013)		1.00	0.64
Imaging visit (2014+)			1.00
**Diabetes**			
No of participants	22,146	738	1416
Baseline	1.00	0.60	0.44
First repeat assessment (2012-2013)		1.00	0.68
Imaging visit (2014+)			1.00

Table S2. Diet component definitions used in the UK Biobank study.

Components	Intake goal	Field IDs	Amount per serving
Fruit	3 servings/day	1309 (pieces fresh fruit/day) 1319 (pieces dried fruit/day)	1309 – 1 piece<br>1319 – 5 pieces
Vegetable	3 servings/day	1289 (tablespoons cooked vegetables/day)<br>1299 (salad/raw vegetables/day)	3 heaped tablespoons
Whole grains	3 servings/day	1438, 1448 (whole meal/wholegrain bread slices/week)<br>1458, 1468 (bran/oat/muesli cereal bowls/week)	1438/1448 – 1 slice/day<br>1458/1468 – 1 bowl/day
Fish	≥2 servings/week	1329 (oily fish/week)<br>1339 (non-oily fish/week)	Once/week
Dairy	2 servings/day	1408 (cheese/week)<br>1418 (milk type)	1408 – 1 piece/day<br>1418 – 1 glass/day if consumption of any type of milk
Vegetable oils	2 servings/day	1428 (Flora Pro-Active/Benecol spread)<br>2654 (Flora Pro-Active/Benecol, soft margarine -, olive oil based -, polyunsaturated/sunflower oil based -, other low/reduced fat spread)<br>1438 (bread slices/week)	1 serving/day if in combination with eating at least 2 slices of bread (ID 1438)
Refined grains	≤2 servings/day	1438, 1448 (white, brown, other bread slices/week)<br>1458, 1468 (biscuit, other cereals/week)	1438/1448 – 1 slice/day<br>1458/1468 – 1 bowl/day
Processed meats	≤1 serving/week	1349 (processed meat/week or daily) 3680 (age when last ate meat)	1349 – 1 piece/day<br>3680 – 0 pieces/day if indicated having never eaten meat
Unprocessed meats	≤2 serving/wk.	1359 (poultry/week or day)<br>1369 (beef/week or day)<br>1379 (lamb or mutton/week or day) 1389 (pork/week or day)<br>3680 (age when last ate meat)	1359-1389 – once/week<br>3680 – 0 pieces/day if indicated having never eaten meat
Sugar-sweetened beverages	Don't drink	6144 (never consumes drinks containing sugar)	0 servings

Field IDs and serving sizes used per diet component in UK Biobank with available data from the general baseline questionnaire. If participants achieved the intake goal they were given 1 point for the diet component.

The total healthy diet score was the sum of all the diet component scores and ranged from 0 to 10.

Table S3. Codes used in the UK Biobank to identify dementia cases and other relevant diseases.

Outcomes	ICD-9	ICD-10	OPCS-4	Self-reported fields	Other
Coronary Heart Disease (CHD)	410, 411, 412, 413, 414	I20-25	K40-K46, K49, K50, K75	6150 (1), 3894, 20004 (1070,1095,1523)	
Diabetes	250	E10-E14	—	2443 (1), 2976, 6153 (3), 6177 (3), 20002 (1220, 1222, 1223)	HbA1c ≥6.5% (48 mmol/mol)
Prediabetes	250 (without diabetes)	E10-E14 (without diabetes)	—	2443 (1), 2976, 6153 (3), 6177 (3), 20002 (1220, 1222, 1223) (without diabetes)	5.7% [39 mmol/mol] ≤HbA1c <6.5% [48 mmol/mol]

Table S4. β coefficients of fish oil use for blood indicators from general linear regression analysis in the UK Biobank in participants with prediabetes and diabetes

	Prediabetes			Diabetes		
**Blood indicator**	**β**	**SE**	**P value**	**β**	**SE**	**P value**
Glucose	0.007	0.009	0.426	-0.075	0.059	0.204
HbA1c	-0.028	0.020	0.157	-0.683	0.234	**0.004**
CRP*	-0.027	0.007	**<0.001**	-0.029	0.011	**0.008**
TG	-0.001	0.011	0.952	0.054	0.020	**0.007**
TC	0.053	0.011	**<0.001**	0.050	0.016	**0.002**
HDL-C	0.009	0.003	**0.009**	0.010	0.005	**0.037**
Large HDL-C	0.002	0.003	0.575	0.004	0.004	0.300
Medium HDL-C	0.001	0.002	0.597	0.005	0.003	0.110
Small HDL-C	0.002	0.001	0.155	0.001	0.002	0.752
Very large HDL-C	0.001	0.001	0.317	0.000	0.001	0.836
IDL-C	0.010	0.004	**0.014**	-0.006	0.006	0.276
LDL-C	0.039	0.009	**<0.001**	0.028	0.012	**0.021**
Large LDL-C	0.019	0.006	**0.001**	-0.011	0.008	0.168
Medium LDL-C	0.007	0.002	**0.005**	-0.006	0.004	0.083
Small LDL-C	0.002	0.001	**0.020**	-0.003	0.001	**0.032**
VLDL-C	0.007	0.005	0.150	-0.012	0.007	0.105
Large VLDL-C	0.001	0.001	0.348	-0.001	0.001	0.386
Medium VLDL-C	0.008	0.004	0.061	-0.006	0.006	0.388
Small VLDL-C	0.003	0.001	**0.011**	-0.002	0.001	0.117
Very large VLDL-C	0.000	0.001	0.917	-0.001	0.001	0.191
Very small VLDL-C	0.003	0.001	**0.008**	-0.001	0.001	0.292
ApoA	0.003	0.003	0.310	0.008	0.004	**0.036**
ApoB	0.013	0.002	**<0.001**	0.009	0.004	**0.013**
IGF-1	0.392	0.060	**<0.001**	0.220	0.105	**0.037**

CRP, C-reactive protein; TG, triglyceride; TC, total cholesterol; HDL, high-density lipoprotein; LDL, low-density lipoprotein

*log (units+1) of CRP values were used.

Results were adjusted for age (continues), sex (male or female), race (white or non-white), assessment centers (22 categories), BMI (in kg/m^2; <18.5, 18.5 to 25, 25 to 30, 30 to 35, ≥35, or missing), Townsend deprivation index (tertiles), household income (<£18,000, £18,000-£30,999, £31,000-£51,999, £52,000-£100,000, >£100,000, or missing), smoking status (never, former, current, or missing), alcohol drinking (never or special occasions only, 1 or 2 times/week, 3 or 4 times/week, ≥5 times/week, or missing), physical activity (in MET-h/wk; tertiles), history of hypertension (yes or no), history of high cholesterol (yes or no), family history of cardiovascular diseases (yes or no), family history of diabetes (yes or no), vitamin supplement use (yes or no), mineral supplement use (yes or no), aspirin use (yes or no), and healthy diet score (tertiles). Further adjusted diabetes duration for diabetic patients (years; <5, 5 to 10, ≥10).

Table S5. Sensitivity analyses for the HRs (95% CIs) of CHD according to fish oil supplement use among participants with prediabetes.

	Fish oil non-users	Fish oil users	P value
**Further adjusting for lipid-lowering drugs**			
No of participants	31,727 (66.6)	15,936 (33.4)	
No of cases (%)	2871 (9.1)	1433 (9.0)	
Age- and sex-adjusted HR (95% CI)	1 [Reference]	0.91 (0.85-0.97)	0.002
Multivariable-adjusted HR (95% CI)	1 [Reference]	0.91 (0.85-0.98)	0.004
**Further adjusting for CRP**			
No of participants	30,169 (66.6)	15,140 (33.4)	
No of cases (%)	2719 (9.0)	1366 (9.0)	
Age- and sex-adjusted HR (95% CI)	1 [Reference]	0.93 (0.87-1.00)	0.038
Multivariable-adjusted HR (95% CI)	1 [Reference]	0.92 (0.86-0.99)	0.029
**Further adjusting for sleep score**			
No of participants	31,727 (66.6)	15,936 (33.4)	
No of cases (%)	2871 (9.1)	1433 (9.0)	
Age- and sex-adjusted HR (95% CI)	1 [Reference]	0.92 (0.86-0.98)	0.007
Multivariable-adjusted HR (95% CI)	1 [Reference]	0.91 (0.85-0.98)	0.011
**Excluding incident CHD within 2 years**			
No of participants	31,309 (66.6)	15,718 (33.4)	
No of cases (%)	2453 (7.8)	1215 (7.7)	
Age- and sex-adjusted HR (95% CI)	1 [Reference]	0.91 (0.84-0.97)	0.005
Multivariable-adjusted HR (95% CI)	1 [Reference]	0.91 (0.85-0.99)	0.019
**Excluding participants who took other supplements**			
No of participants	24,640 (79.1)	6529 (21.0)	
No of cases (%)	2242 (9.1)	595 (9.1)	
Age- and sex-adjusted HR (95% CI)	1 [Reference]	0.90 (0.82-0.99)	0.023
Multivariable-adjusted HR (95% CI)	1 [Reference]	0.92 (0.84-1.01)	0.080
**Excluding participants with missing covariate data**			
No of participants	21,032 (66.5)	10,586 (33.5)	
No of cases (%)	1855 (8.8)	910 (8.6)	
Age- and sex-adjusted HR (95% CI)	1 [Reference]	0.89 (0.82-0.97)	0.006
Multivariable-adjusted HR (95% CI)	1 [Reference]	0.87 (0.80-0.95)	0.002
**Including participants with 5.6 mmol/L ≤ glucose ≤ 6.9 mmol/L)**			
No of participants	38,735 (67.0)	19,122 (33.1)	
No of cases (%)	3479 (9.0)	1708 (8.9)	
Age- and sex-adjusted HR (95% CI)	1 [Reference]	0.90 (0.85-0.95)	<0.001
Multivariable-adjusted HR (95% CI)	1 [Reference]	0.91 (0.85-0.97)	0.003

CI, confidence interval; HR, hazard ratio.

The multivariate model was adjusted for age (continues), sex (male or female), race (white or non-white), assessment centers (22 categories), BMI (in kg/m^2; <18.5, 18.5 to 25, 25 to 30, 30 to 35, ≥35, or missing),

Townsend deprivation index (tertiles), household income (<£18,000, £18,000-£30,999, £31,000-£51,999, £52,000-£100,000, >£100,000, or missing), smoking status (never, former, current, or missing), alcohol drinking (never or special occasions only, 1 or 2 times/week, 3 or 4 times/week, ≥5 times/week, or missing), physical activity (in MET-h/wk; tertiles), history of hypertension (yes or no), history of high cholesterol (yes or no), family history of cardiovascular diseases (yes or no), family history of diabetes (yes or no), vitamin supplement use (yes or no), mineral supplement use (yes or no), aspirin use (yes or no), and healthy diet score (tertiles).

Table S6. Sensitivity analyses for the HRs (95% CIs) of CHD according to fish oil supplement use among participants with diabetes.

	Fish oil non-users	Fish oil users	P value
**Further adjusting for lipid-lowering drugs**			
No of participants	15,836 (71.5)	6310 (28.5)	
No of cases (%)	2393 (15.1)	901 (14.3)	
Age- and sex-adjusted HR (95% CI)	1 [Reference]	0.85 (0.79-0.92)	<0.001
Multivariable-adjusted HR (95% CI)	1 [Reference]	0.88 (0.81-0.95)	0.002
**Further adjusting for glucose-lowering drugs***			
No of participants	15,836 (71.5)	6310 (28.5)	
No of cases (%)	2393 (15.1)	901 (14.3)	
Age- and sex-adjusted HR (95% CI)	1 [Reference]	0.86 (0.80-0.93)	<0.001
Multivariable-adjusted HR (95% CI)	1 [Reference]	0.88 (0.81-0.95)	0.002
**Further adjusting for CRP**			
No of participants	14,773 (71.3)	5941 (28.7)	
No of cases (%)	2247 (15.2)	850 (14.3)	
Age- and sex-adjusted HR (95% CI)	1 [Reference]	0.86 (0.79-0.93)	<0.001
Multivariable-adjusted HR (95% CI)	1 [Reference]	0.88 (0.80-0.95)	0.002
**Further adjusting for sleep score**			
No of participants	15,836 (71.5)	6310 (28.5)	
No of cases (%)	2393 (15.1)	901 (14.3)	
Age- and sex-adjusted HR (95% CI)	1 [Reference]	0.86 (0.79-0.93)	<0.001
Multivariable-adjusted HR (95% CI)	1 [Reference]	0.87 (0.80-0.95)	0.002
**Excluding incident CHD within 2 years**			
No of participants	15,488 (71.5)	6179 (28.5)	
No of cases (%)	2045 (13.2)	770 (12.5)	
Age- and sex-adjusted HR (95% CI)	1 [Reference]	0.86 (0.79-0.93)	<0.001
Multivariable-adjusted HR (95% CI)	1 [Reference]	0.87 (0.80-0.95)	0.003
**Excluding participants who took other supplements**			
No of participants	12,534 (82.1)	2733 (17.9)	
No of cases (%)	1902 (15.2)	394 (14.4)	
Age- and sex-adjusted HR (95% CI)	1 [Reference]	0.83 (0.74-0.93)	0.001
Multivariable-adjusted HR (95% CI)	1 [Reference]	0.86 (0.77-0.96)	0.008
**Excluding participants with missing covariate data**			
No of participants	10,429 (71.3)	4206 (28.7)	
No of cases (%)	1504 (14.4)	608 (14.5)	
Age- and sex-adjusted HR (95% CI)	1 [Reference]	0.91 (0.82-1.00)	0.040
Multivariable-adjusted HR (95% CI)	1 [Reference]	0.92 (0.83-1.02)	0.117
**Excluding participants with type 1 diabetes**			
No of participants	15,602 (71.5)	6222 (28.5)	
No of cases (%)	2359 (15.1)	884 (14.2)	<0.001
Age- and sex-adjusted HR (95% CI)	1 [Reference]	0.85 (0.78-0.92)	
Multivariable-adjusted HR (95% CI)	1 [Reference]	0.87 (0.80-0.95)	0.001

*Glucose-lowering drugs including oral and insulin.

CI, confidence interval; HR, hazard ratio.

The multivariate model was adjusted for age (continues), sex (male or female), race (white or non-white), assessment centers (22 categories), BMI (in kg/m^2; <18.5, 18.5 to 25, 25 to 30, 30 to 35, ≥35, or missing), Townsend deprivation index (tertiles), household income (<£18,000, £18,000-£30,999, £31,000-£51,999, £52,000-£100,000, >£100,000, or missing), smoking status (never, former, current, or missing), alcohol drinking (never or special occasions only, 1 or 2 times/week, 3 or 4 times/week, ≥5 times/week, or missing), physical activity (in MET-h/wk; tertiles), history of hypertension (yes or no), history of high cholesterol (yes or no), family history of cardiovascular diseases (yes or no), family history of diabetes (yes or no), vitamin supplement use (yes or no), mineral supplement use (yes or no), aspirin use (yes or no), and healthy diet score (tertiles). Further adjusted diabetes duration for diabetic patients (years; <5, 5 to 10, ≥10).
